# Evaluation of Asphalt Mixture Low-Temperature Performance in Bending Beam Creep Test

**DOI:** 10.3390/ma11010100

**Published:** 2018-01-10

**Authors:** Marek Pszczola, Mariusz Jaczewski, Dawid Rys, Piotr Jaskula, Cezary Szydlowski

**Affiliations:** Faculty of Civil and Environmental Engineering, Gdansk University of Technology, 80-233 Gdansk, Poland; marjacze@pg.edu.pl (M.J.); dawrys@pg.edu.pl (D.R.); pjask@pg.edu.pl (P.J.); cezary.szydlowski@pg.edu.pl (C.S.)

**Keywords:** low temperatures, bitumen, asphalt mixture, Bending Beam Creep test, BBR test

## Abstract

Low-temperature cracking is one of the most common road pavement distress types in Poland. While bitumen performance can be evaluated in detail using bending beam rheometer (BBR) or dynamic shear rheometer (DSR) tests, none of the normalized test methods gives a comprehensive representation of low-temperature performance of the asphalt mixtures. This article presents the Bending Beam Creep test performed at temperatures from −20 °C to +10 °C in order to evaluate the low-temperature performance of asphalt mixtures. Both validation of the method and its utilization for the assessment of eight types of wearing courses commonly used in Poland were described. The performed test indicated that the source of bitumen and its production process (and not necessarily only bitumen penetration) had a significant impact on the low-temperature performance of the asphalt mixtures, comparable to the impact of binder modification (neat, polymer-modified, highly modified) and the aggregate skeleton used in the mixture (Stone Mastic Asphalt (SMA) vs. Asphalt Concrete (AC)). Obtained Bending Beam Creep test results were compared with the BBR bitumen test. Regression analysis confirmed that performing solely bitumen tests is insufficient for comprehensive low-temperature performance analysis.

## 1. Introduction

### 1.1. Background

One of the most frequent failure types of asphalt pavements that occur at low temperatures are transverse cracks, which are caused by thermal tensile stresses induced under cold winter conditions. Low-temperature cracking starts at the surface of the asphalt pavement and progresses downward with time, due to low winter temperatures as well as rapid drops in ambient temperature. The existence of transverse cracks caused by low temperature leads to other types of degradation of pavement structure. Pavement base, subbase and subgrade can be weakened by water entering the pavement through the cracks.

The general mechanism of the development of low-temperature cracking indicates that cracks occur when thermal tensile stresses exceed the fracture strength of the asphalt pavement layer [1,2,3,4,5,6,7]. Another theory explains low-temperature cracks as an effect of thermal cycling and thermal fatigue failure [8,9]. The number of low-temperature transverse cracks can be reduced by improving the fracture resistance of the asphalt mixtures and by reduction of tensile stresses induced in the asphalt layers. The values of tensile stress in asphalt layers result directly from pavement temperature and viscoelastic properties of asphalt mixtures [10,11]. The viscoelastic properties indicate asphalt mixtures with higher resistance to low-temperature cracking. Therefore, application of adequate test methods for low-temperature performance of asphalt mixtures poses a very important priority in the effort to reduce the occurrence of thermal cracks and it is the main scope of the paper. 

The low-temperature properties of an asphalt mixture seen as a composition of bitumen and aggregate mostly depend on the type of bitumen. The quality of bitumen depends on the bitumen source, which can influence the chemical composition, as well as the production processes used during refining and blending of bitumen [12]. Bitumen is a temperature-susceptible material that becomes soft at high temperatures and harder and brittle at low temperatures. For low-temperature behavior of asphalt binders, there are two leading test methods developed in the USA during the Strategic Highway Research Program (SHRP): the BBR and the Direct Tension Test (DTT) [13,14,15]. The standard BBR test is employed to perform low-temperature creep tests on beams of bitumen conditioned at the desired temperature for one hour. The final result is the limit temperature determined from the stiffness and m-value, which represents the slope of stiffness versus time curve in a double logarithm plot. Both values are determined for the time of loading equal to 60 s. The DTT is used to apply uniaxial tension to a bitumen specimen at a constant strain rate of 3% per minute. As a result, the average stress and strain at failure are obtained. Some studies indicated that the BBR, used to specify asphalt binders, could be also employed to obtain reliable measurements of creep properties of asphalt mixtures [16,17,18,19]. In comparison with standardized BBR method, in the case of asphalt mixtures the time of loading is extended up to 1000 s, and the applied load is properly increased (either 4400 or 6000 mN) [20,21,22,23,24,25,26,27,28]. Since the introduction of the modified BBR method, a significant amount of research has been conducted to determine the low-temperature performance of asphalt mixtures using the scheme of Bending Beam Creep [29,30,31,32,33,34]. The significance of that method of research was also proved by numerical simulations with the use of ABAQUS software [35] and during evaluation of asphalt mixture performance at low temperatures [36,37]. Nevertheless, the effect of mix composition on low-temperature viscoelastic properties of asphalt mixtures still has not been well recognized.

The creep test method in the 3-point bending beam scheme that was presented in the paper can be employed in the following applications:

- Comparative classification of asphalt mixtures by determining stiffness and rheological parameters, including elastic moduli and viscosity coefficients. The viscosity coefficient is directly related to the time of relaxation of thermal stresses at low temperatures. Stiffness, on the other hand, is a parameter directly affecting the amount of generated thermal stresses.
- Determination of advanced characteristics of asphalt mixtures for the purpose of modeling asphalt pavement behavior at low temperatures using thermo-viscoelastic models. The material can be modeled with parameters of the rheological model and/or master curves.
- In combination with the strength of the material, the method can be used to calculate thermal stresses in asphalt pavements and predict the probability of thermal cracking at variable cooling rates.
- Development of functional requirements for asphalt mixtures in the field of resistance to low-temperature cracking according to Construction Products Regulations (in the future).

### 1.2. Objectives

The main objective of the paper is to assess the impact of asphalt mix composition and properties of the asphalt binder on low-temperature viscoelastic properties of the asphalt mixture, which affect the thermal tensile stresses inducing in pavement structure under winter conditions. For this purpose, the master curves of stiffness modulus were analyzed, viscoelastic parameters of asphalt mixtures were evaluated and the relationships between BBR test for bitumen and Bending Beam Creep test for asphalt mixture were determined and discussed.

## 2. Materials and Methods

### 2.1. Materials

#### 2.1.1. Bitumens

Five types of bitumen were selected for low-temperature tests: two neat road bitumens 70/100 and 50/70, two polymer SBS-modified bitumens (PmB) 45/80-55A, 45/80-55B and one highly SBS-modified bitumen 45/80-80. Modified bitumens (PmB) 45/80-55A and 45/80-55B came from two different Polish refineries (Lotos Asfalt, Gdansk, Poland and Orlen Asfalt, Plock, Poland). The standard properties of bitumens used in this research are shown in Table 1.

#### 2.1.2. Asphalt Mixtures

Laboratory tests were conducted on three types of asphalt mixtures: 2 asphalt concretes for wearing courses (AC 11 S for low traffic classes KR1-2, AC 11 S for standard traffic classes KR3-4) and 1 stone mastic asphalt for wearing courses (SMA 11 for medium and high traffic KR3-7). All mixes for wearing courses were designed in compliance with the Polish Technical Guidelines WT-2 2014 [38] and were prepared in the laboratory. The composition of mixtures and types of bitumen used are presented in Table 2. Before the test mixtures were subjected to short-term ageing according to R-28 Standard Specification [39].

### 2.2. Methods

#### 2.2.1. Bending Beam Rheometer Test for Bitumen Testing

Bitumen creep tests were conducted using Bending Beam Rheometer according to the T-313-12 Standard Specification [40]. For each bitumen type, two separate prismatic specimens (102.0 ± 5 mm × 12.7 ± 0.5 mm × 6.25 ± 0.5 mm) were tested at the temperature equal to lower PG + 10 °C. Before the test, each specimen was conditioned at the test temperature for the period of 1 h. The test was conducted for standardized load of 980 mN and the time of loading equal to 240 s. The creep stiffness was calculated on the basis of beam deflections for the time periods of 8.0, 15.0, 30.0, 60.0, 120.0, and 240.0 s using the following equation:

(1) $$S\left(t\right)=\frac{PL^{3}}{4bh^{3}\delta \left(t\right)}$$

where: *S*(*t*)—time-dependent flexural creep stiffness, MPa; *P*—constant load, N; *L*—span length, mm; *b*—width of the beam, mm; *h*—thickness of the beam, mm; *δ*(*t*)—deflection of the beam at the time “*t*”, mm. 

*m*-value was calculated on the basis of binder stiffness using equations:

(2) $$\left|m\left(t\right)\right|=\frac{d\left[logS^{\prime }\left(t\right)\right]}{d\left[log\left(t\right)\right]}=B+2C\left[log\left(t\right)\right]$$

(3) $$logS^{\prime }\left(t\right)=A+B\left[log\left(t\right)\right]+C{\left[log\left(t\right)\right]}^{2}$$

where: *S*’(*t*)—time-dependent flexural creep stiffness estimated using Equation (3), MPa; *t*—time of loading, s; *A*, *B*, *C*—regression coefficients; *S*(*t*)—time-dependent flexural creep stiffness, MPa.

#### 2.2.2. Bending Beam Creep Test for Asphalt Mixtures

The basic procedure of the bending creep test was developed by Judycki [29] and later successively improved [30,41], due to greater availability of more precise and modern equipment. Also, the range of obtained data increased as the method was improved. In this article, the latest modification is presented. While there is a complex state of stresses in the pavement under the influence of low temperatures, the low-temperature cracks are mainly caused by tensile stresses in the asphalt layers. The bending beam creep test chosen for this study was not meant as a direct representation of the stress state, but was used mostly for determination of low-temperature rheological properties of the tested asphalt mixtures. Moreover, the testing method that was applied does not require very sophisticated equipment and may be performed in ordinary road laboratories.

In the test, at least 5 prismatic specimens (50 × 50 × 300 mm^3^) are used for one test temperature. Specimens are sawn from plates (300 × 300 × 50 mm^3^) made of asphalt mixture, compacted using standard laboratory compactor (Cooper Technology, Ripley, UK). The dimensions of prismatic specimens were selected based on applied asphalt mixtures grain size distribution (up to 11 mm) and the literature review [19,23] to reduce the scale effect on the results obtained. The degree of compaction is equal to 99% of Marshall specimen bulk density. The test can be conducted for different temperature sets, depending on the quality of the cooling equipment available in the laboratory. The basic temperature set comprises 4 temperatures: −20 °C, −10 °C, 0 °C and +10 °C. Before the test, each specimen is subjected to a target temperature for 24 h. The curing period was precisely specified in order to avoid the influence of various time-related phenomena, such as physical hardening [42,43,44,45,46,47].

The test is comprised of two main stages. In the first stage, the prismatic specimen is subjected to constant load for 3600 to 10,800 s (dependent on the test temperature) and one linear variable differential transformer (LVDT) transducer (Cooper Technology, Ripley, UK) measures horizontal deformation on the lowest layer of the specimen. In the second stage, the specimen is unloaded, and the LVDT transducer measures the elastic recovery of the specimen. The duration of the first stage was assumed on the basis of previous experience, which showed that for shorter times of loading there are difficulties in determination of rheological parameters. Shorter test times resulted in “diminishing” the steady creep state. Upon extension of the test time, the specimen presented creep, but in a very slow manner.

**Figure 1:**
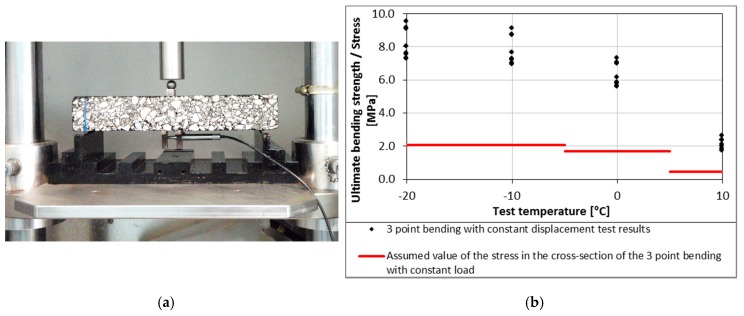
Bending Beam Creep test: (**a**) specimen mounted in the Bending Beam Creep tester; (**b**) exemplary assumed values of the stress during the test.

The level of the test load is determined on the basis of the three point bending test with a constant deformation rate of 1.25 mm/s [48]. For each test temperature, the test load is selected as 30% of the ultimate flexural strength of the weakest tested material in the test series. The aforementioned restrictions were introduced to prevent excessive deformation of the test specimen and to ensure that the results will remain in the linear domain [49,50]. Specimens mounted in the test equipment and assumed levels of load for specific temperatures are presented in Figure 1.

**Figure 2:**
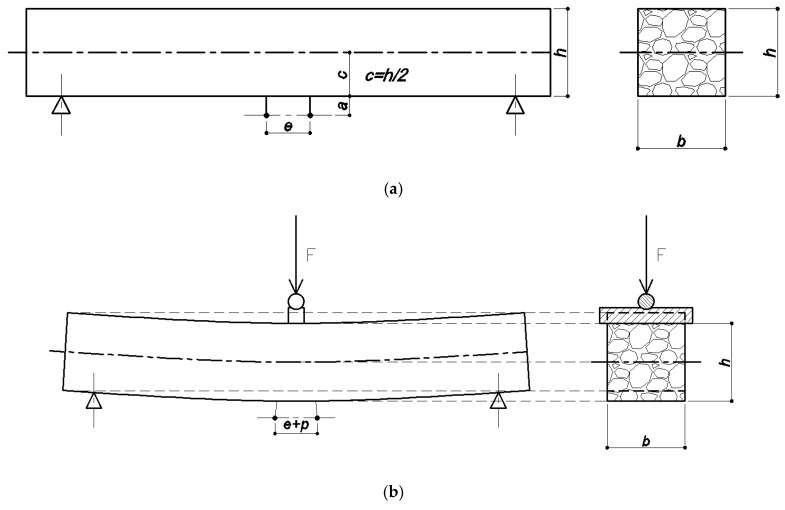
The scheme of the specimen during the test: (**a**) unloaded phase; (**b**) loaded phase.

The following parameters are measured during the whole test: displacement of the LVDT transducer and force applied by the CRT-HYD25 universal test machine (Cooper Technology, Ripley, UK). On the basis of specimen dimensions, the strains and stresses at the bottom of the specimen are calculated using the following equations:

(4) $$\varepsilon =\frac{p}{e}\cdot \frac{c}{c+a}$$

where: *ε*—strain at the bottom of the specimen; *p*—displacement of the LVDT transducer; *e*—the length of the measuring base, mm; *c*—the distance between the center and bottom of the specimen, mm; *a*—the distance between the bottom of the specimen and the center of the LVDT transducer, mm;

(5) $$\sigma =\frac{M}{W}=\frac{3Fl}{2bh^{2}}$$

where: *σ*—stress at the bottom of the specimen in its mid-length, MPa; *M*—bending moment in mid-length of the specimen, kNm; *W*—moment of inertia in the cross section, m^3^; *F*—force applied by the CRT-HYD25 universal test machine, kN; *l*, *h*, *b*—specimen dimensions, mm. All of the described distances and dimensions are presented in Figure 2.

#### 2.2.3. Determination of the Low-Temperature Rheological Properties of Asphalt Mixtures

Rheological properties of asphalt mixtures comprise the main information determined from the Bending Beam Creep test. It is possible to derive at least two sets of data to compare the tested materials or to obtain material properties for further computational analyses. 

Primary usage of the Bending Beam Creep test was to obtain Burgers rheological model parameters, which were later utilized for calculation of thermal stresses. Despite existence of newer models, as yet only the Burgers model has been validated for description of the long-term low-temperature properties [33]. Burgers model parameters are determined using the least square method. For this purpose, each of the creep curves is described using Equation (6), where Burgers model parameters are treated as fitting parameters.

(6) ε(T,t)=σ0·{1E1+tη1+1E2[1−e(−tλ)]}

where: $\lambda =\eta_{2}/E_{2}$, *E*_1_—instantaneous modulus of elasticity, MPa; *E*_2_—modulus of retarded elasticity, MPa; *η*_1_—coefficient of viscosity of steady flow, MPa·s; *η*_2_—coefficient of viscosity of retarded flow, MPa·s; *t*—time of loading, s, *σ*_0_—constant stress during load phase, specific for each temperature, MPa.

**Figure 3:**
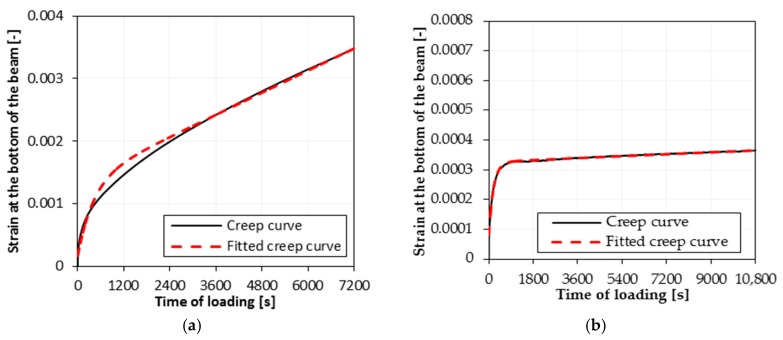
Bending creep test results—creep curves and fitting of the creep using Burgers model at test temperatures: (**a**) 0 °C; (**b**) −20 °C.

Exemplary fittings of the test data for the AC 11S KR3-4 50/70 mixture for selected temperatures are presented in Figure 3.

Secondary material description derived from the Bending Beam Creep test data include the Master Curves of stiffness modulus. The concept of parameterizing the whole spectrum of behavior using one equation was introduced first for polymers [51] and later adapted for bitumen and asphalt mixtures [52]. Currently, numerous models of this concept are extensively utilized for description of viscoelastic behavior of bituminous materials [33,53,54,55,56,57,58]. On the basis of the authors’ experience and a literature review, it was assumed that that the best fitting is obtained with Richards model, which is given by the Equation (7):

(7) log|E*|=δ+α−δ[1+λeβ+γlogf](1λ)

where: *f*—reduced frequency, Hz; *α*, *δ*, *β*, *γ*, *λ*—master curve fitting parameters.

#### 2.2.4. Validation of the Bending Beam Creep Test for Asphalt Mixtures

Previous applications of the Bending Beam Creep test presented major drawbacks in the case of the lowest temperatures, especially in the case of the temperature of −20 °C. For shorter times of loading, equal previously either to 2400 or 3600 s, the creep curve appeared to reach the horizontal asymptote, as in Zener model [51]. A detailed investigation showed that the result was not affected by the equipment. The steady creep was very slow, so it was impossible to derive rheological parameters from the creep curve. From two possible solutions—either an increase in the level of load or an increase in the time of the test, the second option was selected. In the first case there was always the possibility that the measured results could exceed the assumed linear viscoelasticity limits.

**Figure 4:**
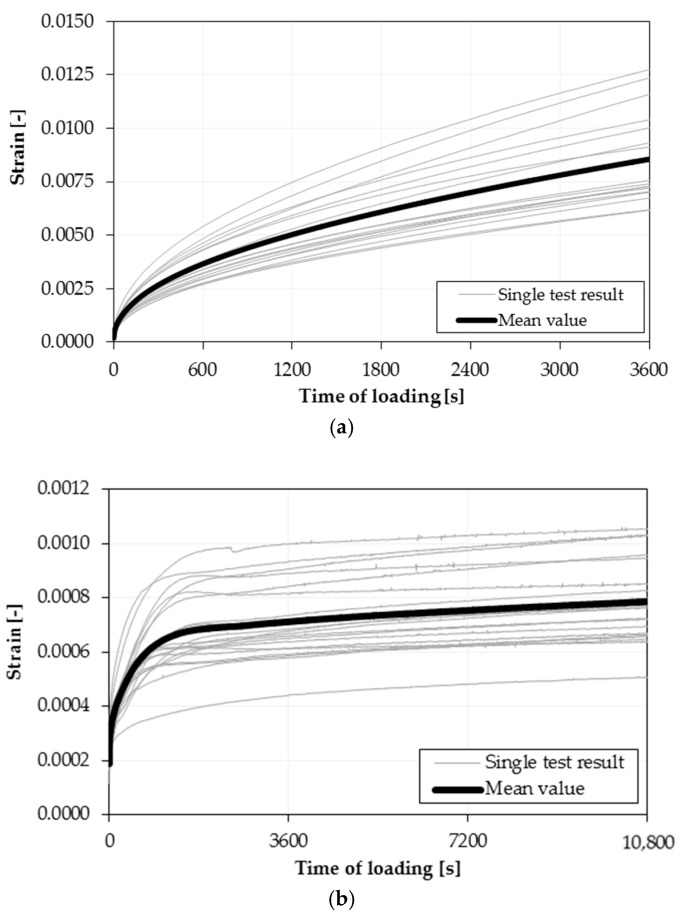
Bending Beam Creep test validation results—creep curves at test temperatures: (**a**) +15 °C; (**b**) −15 °C.

As the methodology and testing equipment were assumed and validated, excessive testing of one selected mixture (typical asphalt concrete for binder course with SBS-modified bitumen) was conducted. To simulate field conditions, the mixture was mixed in the asphalt plant and later compacted in the laboratory. In order to cover a wide range of temperatures using the highest possible number of specimens for each temperature, only three test temperatures were selected: +15 °C, 0 °C and −15 °C. At each temperature, 20 different randomly selected beam specimens were tested. The test load was assumed as 30% of the ultimate flexural strength. To avoid the impact of physical hardening and excessive aging of the specimen, technological periods were assumed as narrow as possible—all specimens were tested from 7 to 13 weeks after compaction, the conditioning time at the target test temperature was assumed as 24 h, and strictly controlled. All test results for the two selected temperatures are presented in Figure 4.

As shown in Figure 4, apart from single creep curves, the test results presented narrow scatter, which is typical of complex materials, such as asphalt mixtures. The coefficient of variation for 20 separate specimens in the case of measured strains is around 20% for both test temperatures. In the case of determined Burgers rheological model parameters, the coefficient of variation is higher and ranges from 10 to 25% for *E*_1_ modulus, from 18 to 40% for *E*_2_ modulus, from 30 to 60% for the *η*_1_ coefficient of viscosity and from 18 to 40% for the *η*_2_ coefficient of viscosity. Nevertheless, the coefficients of variation range from 5 to 25% even in typical tests in which only 4 or 5 separate specimens are tested.

## 3. Results

**Figure 5:**
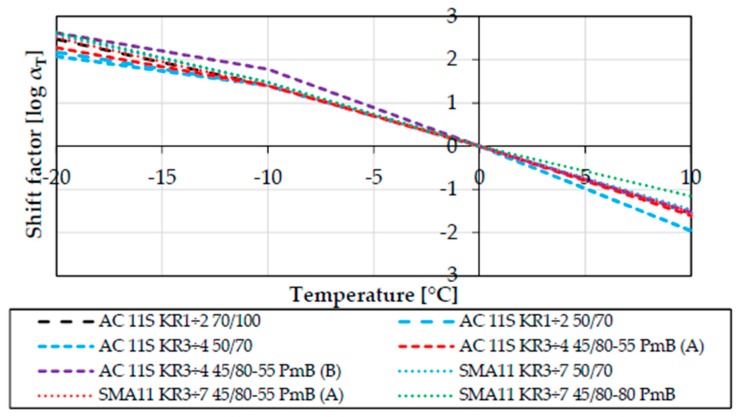
Shift factors for all tested mixtures (reference temperature T_ref_ = 0 °C).

For the purpose of this study, 8 different asphalt mixtures were tested. Mixtures differed both by mixture type and the binder used (neat, polymer-modified, highly modified bitumen). Using the procedure described in Section 2.2.3, Burgers rheological parameters as well as master curve parameters were determined for each mixture, and are presented in Table 3 and Table 4, respectively. Shift factors *α*_T_, additionally derived from the Bending Beam Creep test, are presented in Figure 5.

All presented results are the mean values of the results obtained for five different beam specimens. In the case of master curves, all observed deviations from the time-temperature superposition principle were omitted in the determination of the master curve equation parameters.

Table 5 presents results of the three point bending test with constant deformation rate of 1.25 mm/s conducted at the temperature of −20 °C for all tested mixtures. As was stated in Section 2.2.2, the basic purpose that the data presented in Table 5 served was the determination of load value for the bending creep test. Connecting the results of the two tests—the Bending Beam Creep test and the three point bending test with constant deformation rate conducted in wider range of temperatures—allows us to perform a more detailed analysis of the tested mixtures. For example, it is possible to determine the approximate fracture temperature of the tested mixture or the probability of a fracture event for specific cooling events [10,11].

## 4. Discussion

### 4.1. Analysis of the Burgers Rheological Properties of Aspfalt Mixtures

**Figure 6:**
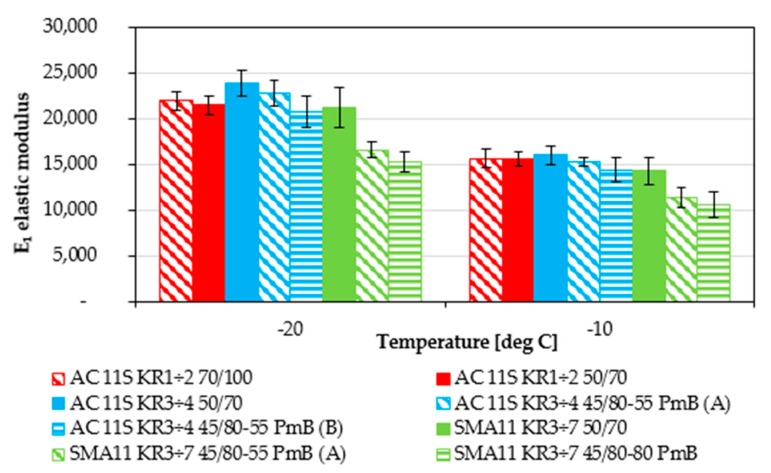
Burgers model E_1_ instantaneous modulus of elasticity results for the temperatures of −20 °C and –10 °C.

**Figure 7:**
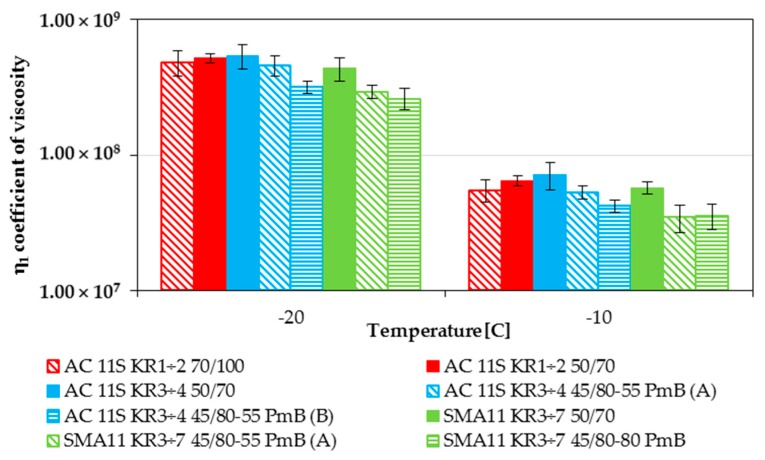
Burgers model *η*_1_ coefficient of viscosity of steady flow results for the temperatures of −20 °C and −10 °C.

From all the determined Burgers model parameters, the two most significant for the low-temperature performance are *E*_1_ instantaneous modulus of elasticity and *η*_1_ coefficient of viscosity of steady flow. The first one is directly connected to the value of thermal stress induced in the asphalt layers by a decrease in temperature. The second one is connected to the relaxation of induced thermal stresses. Values of *E*_1_ modulus determined from the Bending Beam Creep test are presented in Figure 6 and values of the *η*_1_ coefficient of viscosity are presented in Figure 7. All of the presented results are grouped on the basis of mineral mixture used.

An increase in modulus *E*_1_ causes an increase in tensile stresses during cooling of asphalt layer. In the case of *E*_1_ modulus, values obtained for typical asphalt concretes presented similar levels. There were slight differences in the case of asphalt concrete for heavier traffic (KR3-4) at the temperature of −20 °C. Both mixtures with modified bitumen presented lower *E*_1_ stiffness values than the reference mixture with 50/70 neat bitumen. A slight difference was visible between mixtures with polymer modification as well. The mixture with bitumen from refinery B presented better low-temperature performance (lower value of modulus *E*_1_). However, the differences in this case were not significant and lay within the range of scatter of the test results. A more evident situation is visible for stone mastic asphalt mixtures. Mixtures with polymer-modified bitumen presented significantly better low-temperature performance in comparison with the reference mixture with 50/70 neat bitumen. Interestingly and surprisingly, the differences between typical 45/80-55 and highly modified bitumen 45/80-80 were not significant. 

Similar behavior is visible in the case of the *η*_1_ coefficient of viscosity of steady flow. Lower values of *η*_1_ are more desirable because of faster relaxation of the thermal stresses in the asphalt layer. In the case of asphalt concretes, while almost all of them presented the same value, some significant differences are visible, especially in the case of polymer-modified bitumen from the refinery B. In the case of the n_1_ coefficient, AC 11S KR3-4 45/80-55 (B) presented significantly better low-temperature performance in comparison with other mixtures. This is an evident indicator that the basic bitumen tests are not sufficient to predict the low-temperature behavior. As shown in Table 1, in the case of penetration grade both polymer-modified bitumens presented similar values. As for stone mastic asphalt mixtures, their performance was similar to the indications of *E*_1_ modulus. While both mixtures with polymer modification presented significantly better performance in comparison with reference mixture, the mixture with highly modified bitumen was only slightly better than the mixture with typical modified bitumen.

In summary, the most advantageous viscoelastic performance expressed by values *E*_1_ and *η*_1_ was presented by SMA 11 with modified bitumen, and among other mixtures it was on a lower, comparable level. This observation indicates that, beside asphalt binder properties, the mixed composition can improve low-temperature performance of asphalt pavement as well. It can be explained by the thicker film of the bitumen in the SMA type compared with the AC type of mixture.

### 4.2. The Influence of the Mixture and Bitumen Properties on the Low-Temperature Behavior of Asphalt Mixtures

**Figure 8:**
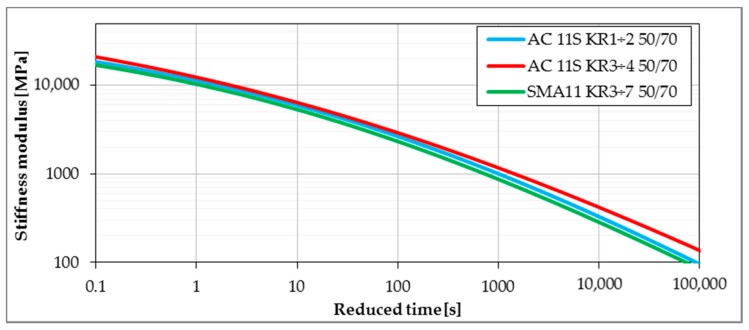
The influence of a mineral skeleton on the stiffness of asphalt mixtures (reference temperature T_ref_ = 0 °C).

**Figure 9:**
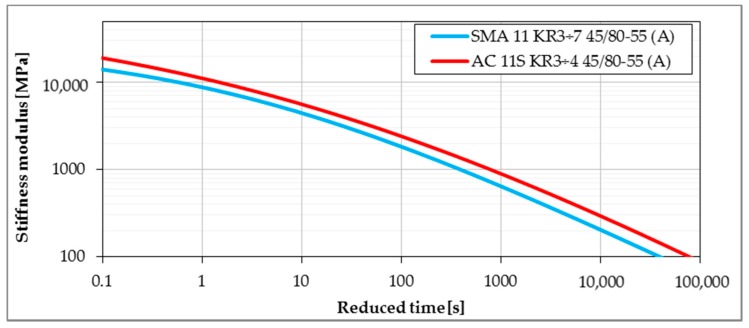
The influence of a mineral skeleton on the stiffness of asphalt mixtures—polymer-modified bitumen (reference temperature T_ref_ = 0 °C).

**Figure 10:**
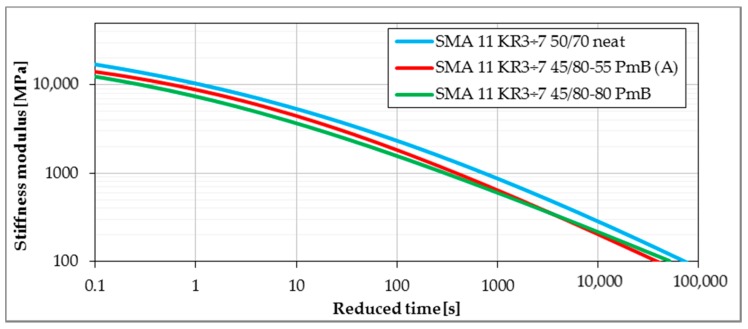
The influence of bitumen type and modification on stiffness of asphalt mixtures (reference temperature T_ref_ = 0 °C).

**Figure 11:**
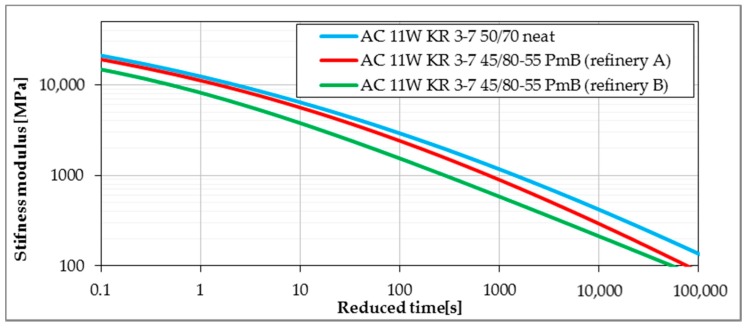
The influence of the base material and modification process on stiffness of asphalt mixtures (reference temperature T_ref_ = 0 °C).

Master curves determined from the Bending Beam Creep test were developed using the procedure given in Section 2.2.3. Lower values of stiffness modulus delivered from master curves express better performance due to lower values of thermal stresses induced in pavement layers. Figure 8, Figure 9, Figure 10 and Figure 11 present the impact of mixture type, bitumen type and content of SBS in modified binder on the values of stiffness moduli in the range of temperatures and time of loading probable under winter conditions. The shift between master curves expresses significance of a given factor (mixture type, bitumen or content of SBS in asphalt binder) on low-temperature performance of asphalt mixture.

As was expected in the case of mixture type, the lowest values of stiffness moduli were obtained for the SMA11 mixture, followed by asphalt concrete for lower traffic. Highest values were obtained for asphalt concrete for heavy traffic. This kind of behavior is strongly connected to the amount of bitumen in the mixture—the higher the amount of bitumen, the stronger the impact on the mixture behavior, and in the result, the lower the value of measured stiffness moduli. 

Also in the case of the type of bitumen, the obtained results present the low-temperature performance in accordance with the expectations. The higher the modification of bitumen, the lower the stiffness modulus at low temperatures. But, similar to the results of the Burgers model rheological parameters, the difference is more visible between neat and modified bitumen. In the case of typical and highly modified bitumen, while the highly modified bitumen presented lower values of stiffness modulus for smaller reduced times (corresponding to lower temperatures), the differences were slight. What is more interesting, for much longer reduced times, the mixture with highly modified bitumen presented higher values of stiffness modulus in comparison with typical modified bitumen, which was probably connected to the lower values of the creep slope obtained for highly modified bitumen at the temperatures of 0 °C and +10 °C.

Unexpectedly, the largest differences in the values of stiffness modulus for all tested mixtures were observed taking into consideration the source of the bitumen. While both modified bitumens presented lower values of the stiffness moduli in comparison with the reference mixture with 50/70 neat bitumen, the difference between bitumen from two different refineries was much larger than in the case of other mixture aspects. This different behavior was probably influenced by the bitumen source and the modification process. As the exact composition of both bitumens was unknown, it could also be assumed that both bitumens probably differed in the amount of the SBS polymer used. The observed difference is more alarming as both commonly used EN and SHRP bitumen tests did not present an evident and significant difference in the low-temperature performance of both tested polymer-modified bitumens. 

### 4.3. Relationship between Results Obtained from Bending Beem Creep Test of Asphalt Mixtures and Asphalt Binders

**Figure 12:**
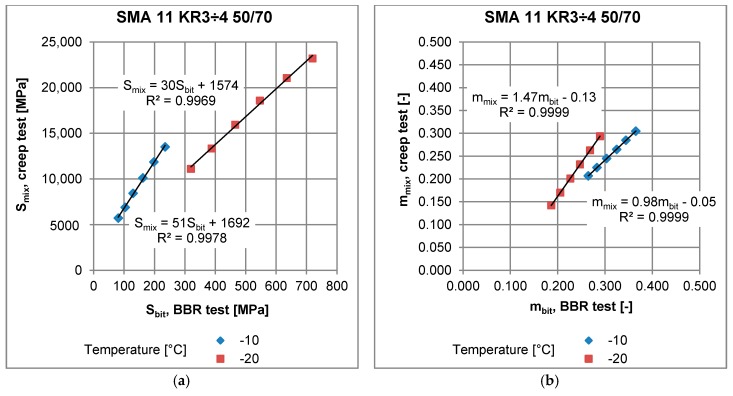
Example of the relationship between (**a**) bitumen stiffness from the BBR test *S_bit_* and stiffness modulus of asphalt mixture from the creep test *S_mix_*; (**b**) m-value of asphalt binder from the BBR test *m_bit_* and *m*-value of asphalt mixture *m_mix_*.

Asphalt mixtures and binders were tested under the same conditions: temperatures −10 °C and −20 °C, and results were obtained for the same times after load application: 8, 15, 30, 60, 120 and 240 s. Both stiffness moduli and m-values of bitumen and asphalt mixtures were determined. Results from the BBR test for bitumen were related to those obtained for asphalt mixtures in the Bending Beam Creep test by means of least squares approximation with linear function. An example of the relationship obtained for stone mastic asphalt SMA 11 with neat bitumen 50/70 is presented in Figure 12. The linear regressions were performed for all tested mixtures listed in Table 6 and yielded a coefficient of determination R^2^ > 0.99 in all cases. This indicates very strong correlation between the results obtained from the creep of bitumen (*S_bit_*, *m_bit_*) and the creep of asphalt mixture (*S_mix_*, *m_mix_*). The relationships are expressed by general formulas:

(8) $$S_{mix}=a_{S}S_{bit}+b_{S}$$

(9) $$m_{mix}=a_{m}m_{bit}+b_{m}$$

where:

*S_mix_*—stiffness modulus of asphalt mixture obtained from the Bending Beam Creep test;
*S_bit_*—stiffness modulus of asphalt binder obtained from the BBR test;
*m_mix_*—slope of creep curve of asphalt mixture obtained from the Bending Beam Creep test;
*m_bit_*—slope of creep curve of asphalt binder obtained from the BBR test;
*a_S_*, *b_S_*, *a_m_*, *b_m_*—regression parameters for a given mixture and test temperature, presented in Table 6.

In the case of the regression obtained for stiffness modulus, an increase in parameters *a* and *b* results in an increase in *S_mix_*, which is an adverse effect due to an increase in thermal stresses in the asphalt layer and a potential increase in the number of low-temperature cracks in the pavement. As for the regression obtained for the *m*-value, an increase in parameters *a* and *b* results in an increase in *m_mix_*, which has a positive effect due to higher potential of stress relaxation of the asphalt mixture. 

The test temperature has an effect on parameters of regression. For all the tested mixtures, a decrease in temperature from −10 to −20 °C caused a decrease in *a_S_* and an increase in *a_m_*, which suggests that the creep parameters of bitumen working within the asphalt mixture are less sensitive to temperature decrease than creep parameters measured for a specimen of asphalt binder.

**Figure 13:**
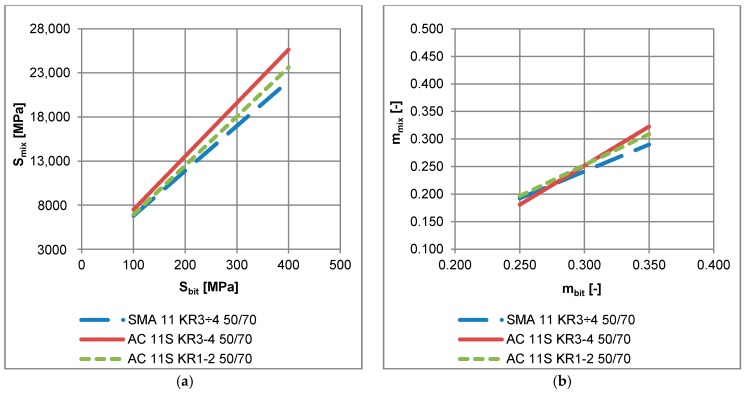
Example of regression functions obtained for three different asphalt mixtures with the same neat bitumen 50/70 (**a**) binder stiffness from the BBR test and stiffness modulus of the asphalt mixture from the creep test; (**b**) *m*-value of asphalt binder from the BBR test and *m*-value of the asphalt mixture.

It was noted that regression performed for the same bitumen applied in different asphalt mixtures yielded different parameters *a_S_* and *a_m_*, which is presented in Figure 13 for neat bitumen 50/70 applied in three different mixtures. These observations indicate that mixture composition has a significant effect on the low-temperature performance of asphalt mixture. Among three asphalt mixtures: AC 11 for low traffic volume KR1-2, AC11 for medium traffic KR3-4 and SMA for medium traffic KR3-4 with the same neat bitumen 50/70, SMA 11 revealed the best low-temperature performance due to lowest values of *S_mix_* (see Figure 13a). On the other hand, it was not possible to assess the low-temperature performance on the base of the *m*-value assessment (Figure 13b). 

**Figure 14:**
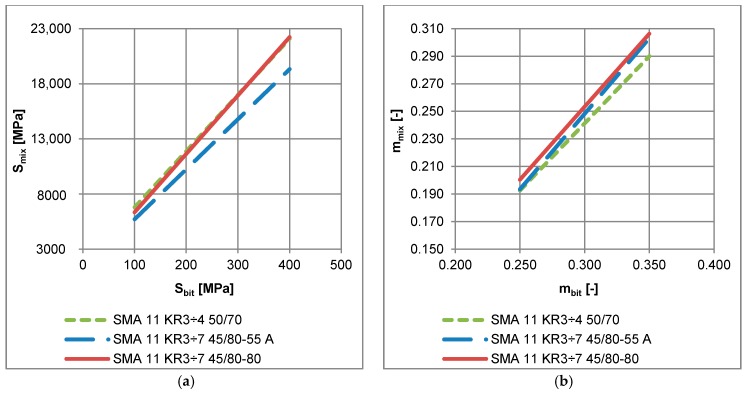
Example of regression functions obtained for one asphalt mixture with different types of bitumen (**a**) binder stiffness from the BBR test and stiffness modulus of the asphalt mixture from the creep test; (**b**) *m*-value of the asphalt binder from the BBR test and *m*-value of the asphalt mixture.

The effect of the application of different types of bitumen in SMA is presented in Figure 14. Regardless of the same mineral composition and bitumen content, the SMA 11 with neat bitumen 50/70 revealed highest values of *S_mix_* and the lowest values of *m_mix_* as a function of *S_bit_* and *m_bit,_* respectively. This indicates the worst low-temperature performance of SMA 11 with neat bitumen 50/70. It should be stated that the usage of the bitumen test method alone is insufficient for assessment of asphalt mixture low-temperature properties, based only on the current standardization of the bitumen binders. This means that the same asphalt binder in different asphalt mixtures may exhibit different low-temperature properties.

## 5. Summary and Conclusions

Based on the test results and analysis, the following conclusions can be drawn:

1. The impact of the asphalt mix composition and properties of the asphalt binder on the low-temperature viscoelastic properties of the asphalt mixture was analyzed and discussed in the paper. The three-point bending beam test of asphalt mixtures was used after detailed validation.
2. The three-point bending beam test of asphalt mixtures was used to determine the low-temperature performance of asphalt mixtures. The performance was evaluated by means of master curves of stiffness modulus, viscoelastic parameters and regression parameters between binder and mixture properties at low temperature. Results obtained from both validation and main test presented highly accurate results. The average coefficient of variation for the validation and main test was in the range from 5 to 25%.
3. The Burgers model parameters *E*_1_, *E*_2_, *η*_1_, *η*_2_ were used to characterize the viscoelastic behavior of asphalt mixtures. An increase in modulus *E*_1_ causes an increase in tensile stresses during cooling of the asphalt layer and contributes to an increase in the number of low-temperature cracks. Lower values of *η*_1_ are more desirable, as they indicate faster relaxation of thermal stresses induced in the asphalt layer.
4. SMA 11 with polymer-modified bitumen 45/80-55 and 45/80-80 presented the best low-temperature performance expressed by the lowest values of *E*_1_ and *η*_1_ in comparison with remaining mixtures: AC 11 with neat and modified bitumens and SMA 11 with neat bitumen 50/70. Promising *η*_1_ values were also presented by the AC 11S mixture with one of the polymer-modified bitumens 45/80-55.
5. The analysis of master curves of stiffness modulus indicates that the source of the bitumen as well as the modification process both have a strong impact on low-temperature performance of the asphalt mixture. While both modified bitumens 45/80-55 presented lower values of the stiffness moduli in comparison with the reference mixture with 50/70 neat bitumen, the difference between bitumens from two different refineries was much larger than in the case of other mixture aspects.
6. Mixture type and the level of modification of the bitumen had an impact on the low-temperature performance of the tested mixtures as well. The best performance was observed for the SMA mixtures. A higher polymer content improved the low-temperature performance as well.
7. The conducted tests showed that the impact of the source of bitumen and its production process (and not necessarily bitumen penetration) on the low-temperature performance of the asphalt mixture is comparable to the impact of mixture type and the impact of polymer content in the binder.
8. Both stiffness moduli and m-values of bitumen and asphalt mixtures were obtained at the same times after load application: 8, 15, 30, 60, 120 and 240 s. Results are given for two test temperatures: −10 °C and −20 °C. The results obtained from the bending beam test for asphalt mixtures are very well correlated with those obtained for asphalt binders from the BBR test with coefficient of determination R^2^ > 0.99.
9. The analysis of viscoelastic parameters, master curves and correlations between binder and mixture stiffness and m-value indicates that the same asphalt binder applied in different asphalt mixtures can provide different low-temperature performances of the asphalt mixture. This indicates that analysis based solely on bitumen testing will not evaluate the low-temperature performance appropriately.
10. The bending beam creep test method can be applied in development of functional requirements for asphalt mixtures in the field of resistance to low-temperature cracking according to Construction Products Regulations (in the future).

The limitations of the study conducted are as follows:

- The test is long and time-consuming; however, previous tests have confirmed that at low temperatures, it is necessary to take into consideration longer load times, such as 10,800 s.
- Sample dimensions and scale effect. Asphalt mixtures with maximum grain size reaching 16 mm and more require a larger sample dimensions.
- There is a complex state of stresses in the pavement under the influence of low temperatures. The bending beam creep test does not directly represent the tensile stresses generated in asphalt mixtures at low temperatures.
- In order to prepare the criteria for asphalt mixture resistance to low-temperature cracking, it is necessary to carry out field assessment and calibration of the method—this subject will be addressed in further research.

## Figures and Tables

**Table 1:** Properties of bitumens.

Property		Type of Bitumen				
		70/100	50/70	45/80-55A	45/80-55B	45/80-80
Penetration at 25 °C, 0.1 mm, acc. to PN-EN 1426	Original	81	54	61	60	53
	RTFOT	48	40	41	40	40
Ring & Ball (R&B) Temperature, °C, acc. to PN-EN 1427	Original	47.8	50.8	63.0	68.6	78.8
	RTFOT	53.4	57.8	61.5	67.4	87.8
Performance Grade, acc. to AASHTO M 320		58–22	64–22	70–22	76–22	82–22
Limit temperature in BBR test, °C,	LST for S = 300	−17	−16	−17	−19	−26
	LmT for m = 0.300	−15	−13	−13	−16	−12

**Table 2:** Properties of asphalt mixtures.

Properties	Type of Mixtures		
Asphalt Mixture	AC 11 S KR1-2	AC 11 S KR3-4	SMA 11 KR3-7
Type of layer	wearing course	wearing course	wearing course
Type of traffic	low	medium	medium and high
Bitumen types	70/100 50/70	50/70 45/80-55A 45/80-55B	50/70 45/80-55A 45/80-80
Binder content (% by mass)	5.8	5.6	6.5
Aggregate type	crushed gravel	crushed granite	crushed granite
Filler type	limestone	limestone	limestone
Sieve size (mm)	% Passing (by mass)		
16	100	100	100
11.2	97	98	95
8	83	77	55
5.6	71	62	39
4	60	52	32
2	40	39	24
0.125	11	11	13
0.063	8	7.2	9.6

**Table 3:** Burgers model parameters (mean values of 5 specimens).

Mixture Type	Bitumen Type	Temp. (°C)	Burgers Model Parameters			
			*E*_1_ (Mpa)	*E*_2_ (Mpa)	*η*_1_ (MPa⋅s)	*η*_2_ (MPa⋅s)
AC 11S KR1-2	50/70	−20	21,982	7859	4.84 × 10^8^	3.54 × 10^6^
		−10	15,655	4735	5.50 × 10^7^	1.40 × 10^6^
		0	10,665	892	3.15 × 10^6^	4.17 × 10^5^
		+10	5819	157	3.98 × 10^5^	4.59 × 10^4^
	70/100	−20	21,531	9987	5.21 × 10^8^	2.81 × 10^6^
		−10	15,582	5421	6.49 × 10^7^	1.49 × 10^6^
		0	10,023	1165	4.35 × 10^6^	5.00 × 10^5^
		+10	4957	220	5.16 × 10^5^	6.49 × 10^4^
AC 11S KR3-4	50/70	−20	23,897	10,032	5.41 × 10^8^	2.53 × 10^6^
		−10	16,009	5719	7.14 × 10^7^	1.75 × 10^6^
		0	10,288	1357	5.34 × 10^6^	5.52 × 10^5^
		+10	5129	183	4.68 × 10^5^	5.80 × 10^4^
	45/80-55 PmB (A)	−20	22,735	8496	4.60 × 10^8^	3.06 × 10^6^
		−10	15,331	4839	5.29 × 10^7^	1.63 × 10^6^
		0	10,325	983	3.85 × 10^6^	5.26 × 10^5^
		+10	5134	174	4.79 × 10^5^	5.40 × 10^4^
	45/80-55 PmB (B)	−20	20,728	9885	3.18 × 10^8^	2.34 × 10^6^
		−10	14,422	3975	4.22 × 10^7^	1.44 × 10^6^
		0	8444	584	2.99 × 10^6^	2.79 × 10^5^
		+10	4576	146	5.34 × 10^5^	4.18 × 10^4^
SMA 11 KR3-7	50/70	−20	21,270	12,022	4.36 × 10^8^	3.58 × 10^6^
		−10	14,291	5326	5.71 × 10^7^	1.65 × 10^6^
		0	8597	980	3.80 × 10^6^	4.52 × 10^5^
		+10	5408	218	4.94 × 10^5^	6.26 × 10^4^
	45/80-55 PmB (A)	−20	16,609	10,080	2.95 × 10^8^	3.35 × 10^6^
		−10	11,332	3700	3.48 × 10^7^	1.44 × 10^6^
		0	8323	698	2.67 × 10^6^	3.55 × 10^5^
		+10	6387	134	3.57 × 10^5^	4.04 × 10^4^
	45/80-80 PmB	−20	15,230	8879	2.62 × 10^8^	3.04 × 10^6^
		−10	10,569	3262	3.57 × 10^7^	1.20 × 10^6^
		0	6454	664	2.89 × 10^6^	2.91 × 10^5^
		+10	4422	218	7.69 × 10^5^	5.82 × 10^4^

**Table 4:** Master Curve parameters (Reference temperature T_ref_ = 0 °C, mean values of 5 specimens).

Mixture Type	Bitumen Type	Richards Model Parameters				
		Max	*β*	*γ*	*δ*	*λ*
AC 11S KR1-2	50/70	4.873	−9.323	−0.329	0.455	0.0004
	70/100	4.745	−8.557	−0.372	0.844	0.0009
AC 11S KR3-4	50/70	5.155	−10.746	−0.271	0.146	0.0001
	45/80-55 PmB (A)	4.929	−10.457	−0.329	0.642	0.0001
	45/80-55 PmB (B)	4.932	−10.382	−0.332	1.211	0.0001
SMA 11 KR3-7	50/70	4.811	−10.931	−0.346	0.741	0.0001
	45/80-55 PmB (A)	4.628	−11.085	−0.399	1.050	0.0001
	45/80-80 PmB	4.694	−10.881	−0.359	1.252	0.0001

**Table 5:** Flexural strength from the three point bending test (test temperature T = −20 °C, mean values of 5 specimens).

Mixture Type	Bitumen Type	Flexural Strength		
		Mean (MPa)	Standard Deviation (MPa)	Coefficient of Variation (%)
AC 11S KR1-2	50/70	7.35	0.81	11.00
	70/100	7.63	0.54	7.13
AC 11S KR3-4	50/70	7.57	0.60	7.92
	45/80-55 PmB (A)	9.19	0.35	3.82
	45/80-55 PmB (B)	9.13	0.54	5.88
SMA 11 KR3-7	50/70	7.29	0.63	8.67
	45/80-55 PmB (A)	8.05	0.84	10.38
	45/80-80 PmB	9.55	1.25	13.08

**Table 6:** Parameters of linear regression of bitumen stiffness and m-values from the BBR test and asphalt mix stiffness from the Bending Beam Creep test.

Type of Asphalt Mixture and Bitumen	Regression Parameters for Stiffness Modulus at Temperature				Regression Parameters for m-Value at Temperature			
	−10 °C		−20 °C		−10 °C		−20 °C	
	*a_S_*	*b_S_*	*a_S_*	*b_S_*	*a_m_*	*b_m_*	*a_m_*	*b_m_*
SMA 11 KR3-4 50/70	51	1692	30	1574	0.98	−0.05	1.47	−0.13
SMA 11 KR3-7 45/80-55 A	45	1177	24	2038	1.10	−0.08	1.66	−0.19
SMA 11 KR3-7 45/80-80	53	1040	29	2101	1.06	−0.06	1.47	−0.15
AC 11S KR1-2 70/100	55	1942	31	1931	0.97	−0.06	2.56	−0.38
AC 11S KR1-2 50/70	56	1370	32	−715	1.12	−0.08	1.90	−0.20
AC 11S KR3-4 50/70	61	1428	37	−1944	1.42	−0.17	2.30	−0.27
AC 11S KR3-4 45/80-55 A	59	1414	37	−928	1.05	−0.06	2.31	−0.29
AC 11S KR3-4 45/80-55 B	65	1292	39	−163	1.17	−0.10	1.86	−0.21
